# Stoichiometric gene-to-reaction associations enhance model-driven analysis performance: Metabolic response to chronic exposure to Aldrin in prostate cancer

**DOI:** 10.1186/s12864-019-5979-4

**Published:** 2019-08-15

**Authors:** Igor Marín de Mas, Laura Torrents, Carmen Bedia, Lars K. Nielsen, Marta Cascante, Romà Tauler

**Affiliations:** 1 The Novo Nordisk Foundation Center for Biosustainability, Technical University of Denmark, Kgs. Lyngby, Denmark; 2 Department of Environmental Chemistry, Institute of Environmental Assessment and Water Research (IDAEA-CSIC), Jordi Girona 18-24, 08034 Barcelona, Spain; 3 Department of Biochemistry and Molecular Biology, Faculty of Biology, Institute of Biomedicine of University of Barcelona (IBUB), Networked Center for Research in Liver and Digestive Diseases (CIBEREHD- CB17/04/00023)) and metabolomics node at INB-Bioinformatics Platform, Instituto de Salud Carlos III (ISCIII, 28029 Madrid), Diagonal 645, 08028 Barcelona, Spain

**Keywords:** Genome-scale metabolic model, Prostate Cancer, Transcriptomic data integration, Stoichiometric gene-protein-reaction association, Endocrine disruptors

## Abstract

**Background:**

Genome-scale metabolic models (GSMM) integrating transcriptomics have been widely used to study cancer metabolism. This integration is achieved through logical rules that describe the association between genes, proteins, and reactions (GPRs). However, current gene-to-reaction formulation lacks the stoichiometry describing the transcript copies necessary to generate an active catalytic unit, which limits our understanding of how genes modulate metabolism. The present work introduces a new state-of-the-art GPR formulation that considers the stoichiometry of the transcripts (S-GPR). As case of concept, this novel gene-to-reaction formulation was applied to investigate the metabolic effects of the chronic exposure to Aldrin, an endocrine disruptor, on DU145 prostate cancer cells. To this aim we integrated the transcriptomic data from Aldrin-exposed and non-exposed DU145 cells through S-GPR or GPR into a human GSMM by applying different constraint-based-methods.

**Results:**

Our study revealed a significant improvement of metabolite consumption/production predictions when S-GPRs are implemented. Furthermore, our computational analysis unveiled important alterations in carnitine shuttle and prostaglandine biosynthesis in Aldrin-exposed DU145 cells that is supported by bibliographic evidences of enhanced malignant phenotype.

**Conclusions:**

The method developed in this work enables a more accurate integration of gene expression data into model-driven methods. Thus, the presented approach is conceptually new and paves the way for more in-depth studies of aberrant cancer metabolism and other diseases with strong metabolic component with important environmental and clinical implications.

**Electronic supplementary material:**

The online version of this article (10.1186/s12864-019-5979-4) contains supplementary material, which is available to authorized users.

## Background

Cancer is influenced by genetic and environmental factors and is one of the leading causes of death worldwide [1]. The multi-factorial nature of this disease represents a challenge for diagnostic approaches and imposes a high burden on health care systems [2].

Genome-scale metabolic models (GSMM) have emerged as a potential tool to decipher the molecular mechanisms underlying cancer metabolism associated with tumor progression and malignancy acquisition [3]. GSMMs are built in a bottom-up manner gathering all known biochemical reactions encoded by a given organism’s genome [4]. Additionally, these models describe the associations between genes, proteins and reactions (the so-called GPRs) [5]. GPRs are generated using Boolean formulations describing gene(s) encoding the protein(s) required to catalyze a given reaction.

GPRs enable the integration of gene or protein expression data from different high-throughput techniques which enhances the predictive capabilities of GSMM-based analysis. A large number of computational approaches have been developed to integrate expression data through GPRs into constraint-based methods [6]. However, current GPR formulations do not take into account the number of protomers required to generate a fully functional catalytic unit. Thus, the lack of stoichiometry represents an important limitation in transcriptomics-based model-driven methods to study the metabolism. This drawback reduces the scope of these approaches to study multi-factorial diseases in complex scenarios. Hence, in order to overcome these limitations, we present a new state-of-the-art GPR: Stoichiometric GPR (S-GPR). Here, we include information about the transcript copy number required to produce all the subunits of a fully functional catalytic unit capable to carry metabolic flux through a given reaction/s.

As a case of concept, we delved into the effect of chronic exposure to Aldrin, an endocrine disruptor (ED), in DU145 prostate cancer (PC) cells. This endocrine disruptor can be found at low concentrations in the environment [7]. While many pieces of evidence suggest a dose-effect relation between EDs and enhanced tumor malignancy, our understanding of the effects of chronic exposure to non-lethal concentrations of EDs in cancer metabolism remains limited. However, it has been reported that long-term exposure to Aldrin produces important alterations in the metabolic and lipidomic profile of PC cells, which are associated with an increased tumor malignancy [8]. Apart of the biological and environmental interest, this case presents important technical challenges from the modeling and data integration point of view due to the high similarity of the transcriptomic profile between Aldrin exposed and non-exposed cells. Here, only 0,37% of the genes are significantly different between conditions (*p* < 0,01), among which only 1,6% are metabolic genes (Additional file 3). Thus, inferring metabolic alterations based on the gene expression by applying transcriptome-based model-driven approaches is especially challenging in this case. On the other hand, test the S-GPR formulation on a more coarse-graining case, with more significant differences between condition may disguise the effect of incorporating the stoichiometry to the gene to reaction formulation. For example, in case of having a complex with multiples sub-units encoded by the same gene, incorporating the stoichiometry into the gene-to-reaction formulation may not provide further improvements. Due to the difference between conditions is too high, the gene will unlikely become the limiting factor at generating a functional catalytic unit regardless stoichiometry is considered or not.

In the current study, we evaluated the improvements in transcriptomics-based model-driven analysis, when the stoichiometry is incorporated to the classical GPR formulation (S-GPR), to study the metabolism of Aldrin-exposed and non-exposed DU145 PC cells. It was achieved by integrating the transcriptomic data through GPR and S-GPR rules into one of the latest human GSMM: HMR2 [9]. It was performed in different steps: i) build both GPR and S-GPR rules based on different data bases [10–12], ii) next, enrich the GSMM to account for all the experimentally measured lipids and metabolites, iii) predict metabolic consumption/production by integrating transcriptomic data from Aldrin-exposed and non-exposed cells via GPR and S-GPR into a GSMM reconstruction analysis using some of the most widely used constraint-based methods and iv) compare the predicted consumption/production of metabolites and lipids with experimental measurements.

Significant improvements in the predictive capabilities of the GSMM have been observed when S-GPRs were implemented, correcting some wrong predictions provided by GPR-based approaches.

Furthermore, all the analyses performed in this study revealed important alterations in key metabolic pathways associated to the chronic exposure to Aldrin. Thus, prostaglandin biosynthesis was predicted to be over-activated in Aldrin-exposed cells, which is associated with malignancy acquisition [13, 14]. Creatine shuttle was also predicted to be over-activated in Aldrin-exposed cells. Long-chain fatty acids are actively transported through this shuttle and its over-activation is associated with tumor progression and malignancy acquisition [15, 16].

The approach presented here represents an important step forward in the state-of-the-art of the current transcriptomic-based model-driven methods and opens new avenues in the study of the molecular mechanisms underlying multi-factorial diseases in complex scenarios such as the case study presented here, with potential clinical and environmental applications.

## Results

### Refinement of the generic GSMM to fit specific context features

The computational analysis performed in this work was based in one of the latest reconstruction of human metabolism (HMR2) [17], with 136 metabolic pathways, 3160 unique metabolites, 3765 metabolic genes and 8181 reactions. The model was modified to fit with our case study. Specifically, the model was expanded to include all the exchange reactions of the experimentally analyzed metabolites and lipids that were already annotated in the model. These reactions can be interpreted as the way the model can uptake or release a given metabolite if the respective exchange reaction carries non-zero flux in either forward or backward direction, which can be compared with the experimental measurements to determine the reliability of model predictions. Additionally, the model was reduced by removing blocked reactions and dead-end metabolites (Methods section). Gene-to-reaction associations were also included in the GSMM, so the transcriptomic data could be integrated in the model. The resultant GSMM had 134 metabolic pathways, 2339 unique metabolites, 3096 metabolic genes and 6941 reactions. This process is shown in more detail in the Additional file 4.

### Reliability of the gene-to-reaction building

The gene-to-reaction associations included in the HMR2 were automatically build by using an in-house developed algorithm that gathers information retrieved from a number of databases to generate both GPRs and S-GPRs (Additional file 5) [10–12].

Here, we evaluate the reliability of the automatically-built gene-to-reaction associations with the ones from a published model. The evaluation was done by comparing experimental measurements with the metabolic exchange rates predicted by HMR2 model with automatically-built GPRs (hereinafter HMR2 + GPR) and by Recon 2 (which incorporates GPRs) [18]. To this aim transcriptomic data from Aldrin-exposed and non-exposed cells were integrated into both GSMMs by applying the iMat algorithm [19]. This approach is based on transcriptomic data to define an objective function that maximizes the similarity between gene expression and the activity state of the metabolic reactions. Since the iMat algorithm only uses transcriptomic data to impose additive constraint to FBA solutions, this approach is well suited to test the reliability of our automatically-built GPRs at integrating transcriptomic data into GSMMs. Since HMR2 accounts for more metabolite exchange reactions than Recon 2, the evaluation was based in the proportion of right predictions (right predictions/(right predictions + wrong predictions)) rather than in the absolute number of right prediction. Our computational analyses show that the 60.6% of the prediction provided by Recon2 were in accordance with experimental observations, while in HMR2 incorporating conventional GPRs built by our algorithm reached the 79.3%. These results indicate that the automatically-built gene-to-reaction associations enable a proper integration of transcriptomic data into genome-scale metabolic model reconstructions analyses. The process and results are shown in more detail in the Additional files 9 and 7 respectively.

### Improving model predictions by implementing S-GPRs

**Fig. 1:**
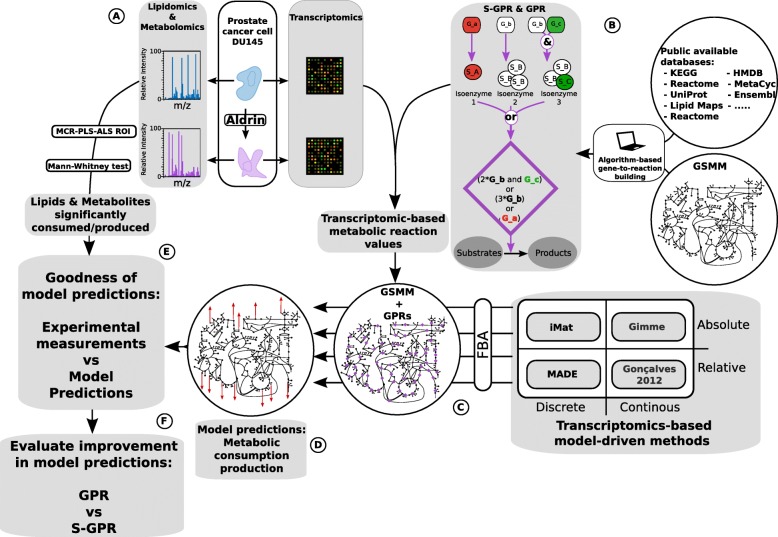
Work-flow overview. **a**. Experimental data acquisition from Aldrin-exposed and non-exposed DU145 prostate cancer cells. Relevant peak areas from non-targeted metabolomic an lipidomic experiments are set by applying MCR-ALS and ROI methods, next significant consumption/productions are determined through Mann-whitney test **b**. Algorithm-based automatic gene-to-reaction association building. The algorithm uses a variety of data bases to generate a set of gene-to-protein associations for each reaction with enzymatic activity in a model. **c**. transcriptomic data integration via either GPR and S-GPR into a GSMM reconstruction analysis by applying four different constraint-based methods **d**. model prediction of metabolic consumption/production **e**. Validation of the prediction by comparing predicted and experimental metabolic consumption/production (Fisher exact test) **f**. Evaluate the improvement in model predictions provided by the incorporation of stoichiometry into the gene-to-reaction associations (S-GPR)

**Fig. 2:**
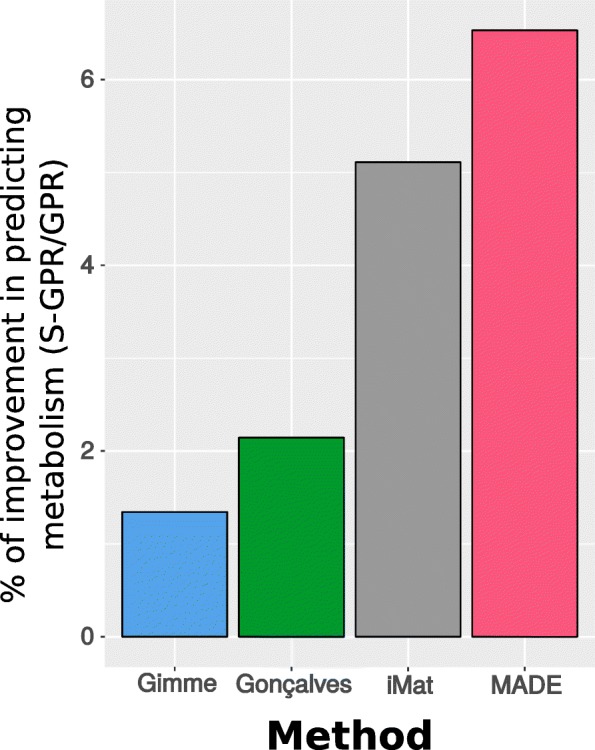
Metabolic consumption/production predictions S-GPR vs GPR. Percentage of improvement in predicting reaction activity in each method using S-GPR compared with GPR. Significance tested with Fisher’s Exact test [23] with *p* < 0.05 in all the analyses

Adding stoichiometry to the classical gene-to-reaction formulation (S-GPR) enable us to consider the copy number needed to generate the active catalytic unit of an enzyme. In order to evaluate the improvement in GSMMs at predicting metabolism when S-GPRs are implemented, different methods for integrating transcriptomic data were used on HMR2 model incorporating either GPRs or S-GPRs: Gimme, iMat, Gonçalves et al. 2012 and MADE [20, 22] (see Methods and Fig. 1C). In both Gimme and iMat, thresholds were used to classify reactions as associated to highly-expressed or lowly-expressed genes. In our analysis these thresholds were set at 40th and 60th percentiles as lower and upper thresholds respectively in iMat and at the mean + standard deviation in Gimme. The chosen thresholds were those that provided the highest discrimination between active and inactive reactions between conditions (Additional files 8 and 9). The method described by Gonçalves et al. 2012 does not require a predefined set of parameters. Finally, the MADE algorithm differentiates the active and inactive reactions based on the fold change between conditions and the associated *p*-value. The parameters and the procedure used to evaluate the chosen thresholds allowed us to determine which threshold was better to discriminate between active and inactive reactions taking into account the transcriptomic data sets. From these analyses, a set of fluxes were obtained from which the exchange of the different species could be inferred. Specifically, we analyzed the activity state of the exchange reactions for the species experimentally measured (active reaction, if there is non-zero flux through either forward or backward direction, otherwise inactive) and qualitatively compared with the experimental measurements (consumption or secretion active if a significant difference in metabolite concentration between t_0_ and t_5h_ is observed, otherwise inactive) to determine the reliability of the model predictions. The exchanges predicted from each method were qualitatively compared with experimental measurements of metabolic and lipidomic data and its significance was determined by Fisher’s Exact test using α = 0.05 (Additional file 8). This analysis demonstrated an improvement in terms of correct predictions when S-GPRs were implemented compared with the classical GPRs. The improvement in the number of correct predictions varied among the four methods from 1.34% using Gimme to 6.53% using MADE (Fig. 2).

In all the tested methods, the S-GPR implementation corrects some metabolic uptakes/secretions predicted by classical GPR-based analyses that were not supported by experimental measurements (Table 1). For instance, the Gimme algorithm based on GPRs failed at predicting glycocholate exchange in both Aldrin-exposed and non-exposed cells. The MADE algorithm using GPRs also failed at predicting pantethiene exchange in both cell groups. In both cases the same algorithms correctly predicted the experimental observations when S-GPRs were implemented. Interestingly, alterations in glycocholate and pantethiene levels have been associated with malignancy acquisition in different types of cancer [24, 25], which supports the predictions provided by the S-GPR-based analyses and highlights the importance of incorporating stoichiometry into transcriptome-based model-driven analyses. This is shown in Table 1 and in more detail in Additional file 8.

**Table 1:**
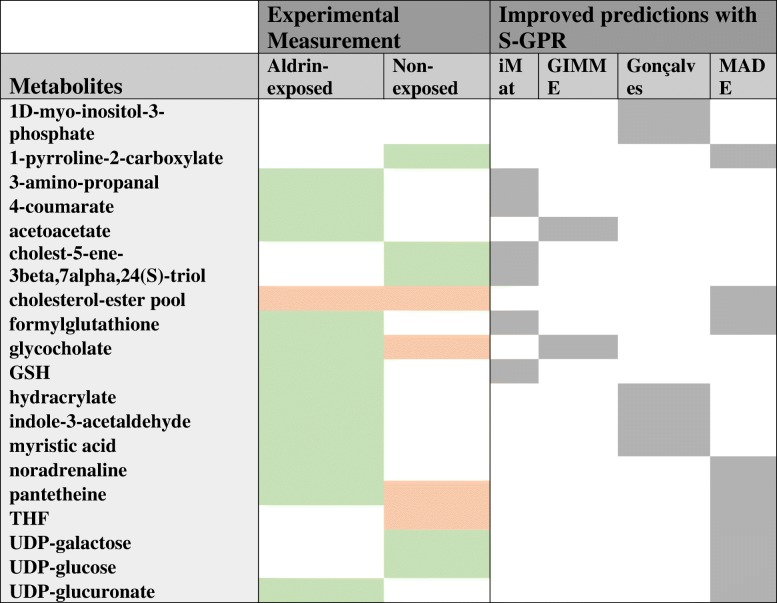
Metabolic consumption/production perditions S-GPR vs GPR

Metabolites’ uptake/secretion that have been wrongly predicted by GPR-based analyses and corrected when applying S-GPRs. In the first column are represented the metabolites. Column 2 and 3 show the significant metabolites’ uptake/secretion experimentally measured in Aldrin-exposed and non-exposed DU145 cells respectively. Here green and red represents either metabolite consumption or production respectively. The four last columns represent the four different methods used to integrate transcriptomic data into a GSMM reconstruction analysis. Here, cells highlighted in gray represents those cases in which GPR-based analyses provided a wrong prediction that was corrected when using S-GPR instead (the opposite case haven’t been observed in our case study)

### Inferring alterations in metabolic pathways associated to chronic exposure to Aldrin

**Fig. 3:**
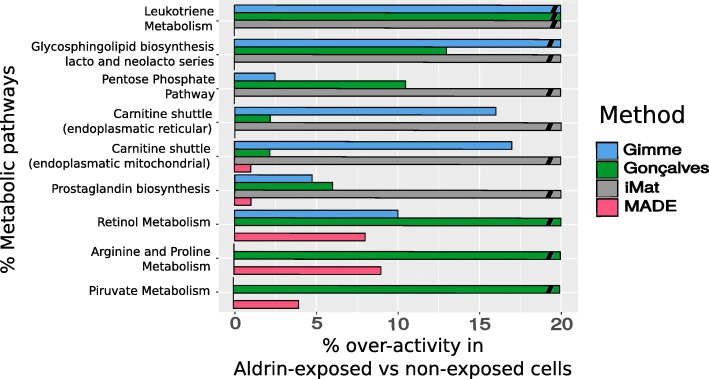
Pathways over-activation predicted in each method. Bars represent the % of pathway over-activity in Aldrin-exposed cells compared with non-exposed cells (n° of active reactions in Aldrin-exposed cells/n° of active reactions in non-exposed cells). The non-continuous bars indicate that a given pathway is predicted to be active only in Aldrin-exposed cells. Its significance was tested with a t-test [28] (*p*-value < 0.05)

From each of the four transcriptomics-based model-driven analyses performed in this study, active and inactive reactions were predicted. Based on these results we determined which pathways were over-activated in one condition compared to the other. Pathways showing a significantly higher number of active reactions in Aldrin-exposed analysis compared to non-exposed were tracked down as indicated in the Methods section. Nine overactive pathways were identified in at least three of the four methods, including two predicted to be over-activated in Aldrin-exposed cells by all four analyses: Prostaglandin Pathway and Carnitine Shuttle. In Fig. 3 all nine pathways are indicated with the relative weight in Aldrin-expressed analysis with S-GPR. The relative weight was calculated as follows: (number of active reactions of the pathway in Aldrin-exposed cells) / (number of active reactions of the pathway in non-exposed cells).

## Discussion

High-throughput technologies have transformed molecular biology into a data-rich discipline. In order to extract knowledge from this large amount of data, a number of approaches have been developed. Among these methods, those integrating transcriptomic data into GSMM have emerged as potent tools, since they enable us to determine molecular processes involved in a specific case from one of the most widely used and cost-effective high-throughput techniques. However, these approaches present important limitations when trying to deal with complex scenarios as the case studied here.

The present work aimed to improve the predictive capabilities of the current transcriptomic-based model-driven approaches by incorporating stoichiometry to the GPR rules embedded in GSMM. For illustration, we have studied the effects of the chronic exposure to Aldrin on DU145 PC cells metabolism. The multi-factorial nature of cancer disease together with the complexity of this study, which goes beyond the classical dose-effect studies, increases the difficulty of determining metabolic changes associated with an increase of tumor malignancy in Aldrin-exposed cells. While the human genome microarray platform HG U-219 used for transcriptomics analysis covers more than 36,000 transcripts and variants (representing 20,000 genes), comparing gene expression data obtained from Aldrin-exposed and non-exposed cells identified only 0.35% of difference between conditions. In such complex scenarios, the implementation of S-GPRs has improved model performance at predicting metabolite and lipids exchange in all the methods tested (from 1.34% up to 6.53%). This improvement has been accompanied by changes in the overall activity state of the metabolic network (from 1,79% up to 3,57%) (Additional file 8). Additionally, the implementation of S-GPR into transcriptomic-based model-driven methods has corrected several wrongly predicted exchanges generated by classical GPR-based analyses (Fig. 2 and represented in more detail in Additional file 8). The degree of improvement was significant in all the analyses when S-GPRs were implemented and it is especially relevant since the transcriptomic profile of non-exposed and Aldrin-exposed cells differs in only 0.35%.

The improvement was achieved despite using transcriptomics as a proxy for proteomics. While transcript level correlates with protein levels, the correlation is relatively poor [26]. It would be expected that the performance would be further improved either when using proteomics or if the translational efficiency for each protein was included [27].

Furthermore, a pathway analysis of the results provided by the different approaches when S-GPRs were implemented revealed an over-activation of different pathways (Additional file 9). The different approaches used to integrate transcriptomic data into GSMM reconstruction analysis provided very similar results. For instance, Leukotriene metabolism was predicted to be exclusively active in Aldrin-exposed cells by all the approaches except by MADE. In general, iMat and Gonçalves et al. 2012 approaches tend to predict a complete inactivation of certain pathways in non-exposed cells. Thus, apart of the above-mentioned Leukotriene pathway, five and three non-overlapped pathways were predicted to be active exclusively in Aldrin-exposed cell in iMat and Gonçalves et al. respectively. The GIMME algorithm identifies only two pathways exclusively active in Aldrin-exposed cells, while MADE algorithm, in general, predicts less differences between condition and in any case pathways only active in Aldrin-exposed cells. These results highlight the different predictive capabilities of the different methods and their sensitivity to gene expression differences. However, two key pathways were identified as over-activated in Aldrin-expressed cells across all the methods: Carnitine shuttle to mitochondria and prostaglandin biosynthesis (Fig. 3) with relevance for tumor proliferation, invasiveness, and metastasis. In this sense, prostaglandin metabolism has already been related to the regulation of mechanisms associated to malignancy [13]. Interestingly, Marin de Mas et al. already reported in 2018 the relevance of these pathways in the Epithelial-mesenchimal-transition programme (EMT) in the PC-3 prostate cancer cells as well as the importance of the long-chain fatty acids in the process [16]. More specifically, prostaglandins have been found to be related to an increase of the angiogenic capacity of the tumor [29]. Conversely, the Carnitine shuttle pathway involves numerous cytosolic substrates among which there are some long-chain fatty acids with antiproliferative effects [15]. Thus, a higher activity of this pathway in Aldrin-exposed cells may have two roles: i) first, and probably the most evident, fuel mitochondrial beta-oxidation to maintain the energetic requirements imposed by a higher proliferation rate and ii) to eliminate compounds with antiproliferative effects. Secondly, prostaglandin metabolism produces a variety of molecules such as 2S-HETE, TXB2 and PGE2 which promote cell adhesion, angiogenesis, and cell invasion in prostate cancer [13]. In addition EP4, one of the PGE2 receptors, has been recently investigated as a potential immune-oncology drug target [30]. Despite some prostaglandines and fatty acid associated to the carnitine shuttle have been detected, no significant differences in the consumption/secretion rates between condition have be determined. It can be due a low of sensitivity to measure lowly abundant species of the non-targeted metabolic approach used in this study. Thus, more sensitive targeted approaches would be required in order to further investigate how the chronic exposure to Aldrin alters prostaglandine and carnitine shuttle pathways and the role in the adquisition of malignancy in PC DU145 cells. Overall, the model predictions were consistent with reported evidences and experimental phenotypic observations.

Although these findings are supported by the four different methods and are consistent with reported evidences, it is necessary to consider that solutions provided by CBM are not unique. This can be further tested by applying a robustness test and a sensitivity analysis on the methods used here, which will provides the list of reactions that are unambiguously active or inactive in a particular conditions. Finally, model predictions would need to be further validated experimentally.

## Conclusion

The current work highlights the improvement of GSMMs predictive capabilities when stoichiometry is incorporated into gene-to-reaction associations (S-GPR). This novel approach has been tested with different methods to integrate transcriptomic data into GSMM reconstruction analyses with the same result: S-GPR incorporation improves model analysis performance. In addition, this novel concept has been tested on a very complex scenario involving a multifactorial disease, activation of secondary metabolism triggered by very low concentrations of pollutant and two highly isogenic populations which make more relevant the improvements provided by S-GPR based analyses. Despite it does not exist a lineal relationship between individual gene expression and specific metabolic flux, the incorporation of stoichiometry into the gene-to-reaction formulations has enhanced the predictive capabilities of the transcriptome-based model-driven approaches used in this study at evaluating the effects of the chronic exposure to Aldrin in DU145 PC cells. The approach presented here has the potential to be extrapolated to the study of other cancer types like the NCI-60 cancer cell lines of which consumption/release metabolic profiles and gene expression can be retrieved from the CORE database [31]. Thus, the approach we are introducing here is conceptually new and its results highlight the importance of our proposal, which paves the way for more accurate analyses with possible important environmental and clinical implications.

## Methods

### Omic datasets preprocessing

Non-targeted metabolomic data, lipidomic data and cDNA samples for transcriptomics analysis were obtained from a previous study [8] which tackled the lipid profile alterations in experiments of chronic exposure of DU145 PC cells to Aldrin, an ED. Sampling of Aldrin-exposed and non-exposed cells and measurements were conducted after 50-days-long exposure [8]. Following, it is explained in more details how the metabolomic, lipidomic and transcriptomic data were pre-treated before being used in our computational analysis.

Metabolomic and lipidomic data analysis: In this work, lipid and metabolite measurements from non-targeted LC–MS analysis at time 0 and after 5 h of incubation were used. Lipidomic data was generated by following the same protocol of lipid extraction and analysis as Bedia et al., 2015. Metabolomic data was obtained as follows: metabolites from snap**-**frozen cells were extracted using cold methanol/chloroform (90:10). After vortexing, mixtures were centrifuged 5 min at 16000 g. The supernatants were evaporated and further dissolved in 150 μl of LC–MS mobile phase [32]. The LC–MS analysis was carried out using a LC–ESI–HRMS, Orbitrap Exactive HCD (Thermo) with a HILIC TSK Gel Amide-80 column (250 × 2.1 mm, 5 μm particle size, Tosoh Bioscience). Elution gradient was performed using solvent A (acetonitrile) and solvent B (5 mM of ammonium acetate adjusted to pH 5.5 with acetic acid) as follows: 0–8 min, linear gradient from 25 to 30% B; 8–10 min, from 30 to 60% B; 10–14 min, 60% B; 14–20 min, back linearly from 60 to 25% B; and from 20 to 27 min, 25% B.

The resulting chromatogram matrix was compressed by applying regio-of-interest (ROI) strategy [33] together with multivariate curve resolution alternating least squares (MCR-ALS) [34], as previously described in Marques A.S. et al. 2016 [35]. In brief, ROI approach imposes multiple criteria to compress the data matrix with no loss of information. The used criteria includes: i) a signal-to-noise ratio threshold (STNRT) above which a given signal is considered as relevant, ii) a minimum number of consecutive retention times above the STNRT and iii) mass accuracy of the mass spectrometer (Additional file 1).

Next, the relevant molecular masses and retention times of the lipids and metabolites annotated in the model were identified by using both home-made and external on-line databases (Human Metabolome Database [36], Lipid Maps [37] and Human metabolic atlas [38]). As a result, we were able to determine the abundance of 75 lipids and 169 metabolites at each time point (Additional file 2). Following, the metabolite and lipid consumption/productions were determined by comparing the peak areas at time 0 and after 5 h in both Aldrin-exposed and non-exposed cells (Fig. 1A). The significance between the two time-points was determined by Mann-Whitney test [39]. Thus, for each measured metabolite we qualitatively determine whether it was significantly consumed/secreted or not. Both, lipidomic and metabolomic data were used to determine the reliability of the model predictions.

Transcriptomic data analysis: The gene expression profiles from Aldrin-exposed and non-exposed DU145 cells were obtained by using HG-U219 array plate (Affymetrix inc. California, USA), after 50 days of exposure to sub-lethal concentration of Aldrin that did not affect the proliferation rate of tumoral cells. Microarray data was normalized by using RMA method [40] (Additional file 3) (Fig. 1A). Transcriptomic data was integrated into the genome-scale metabolic network reconstruction analysis by applying different computational approaches (Fig. 1C) [20, 22].

### GSMM readjustments/refinement

Genome-scale metabolic model: The computational analyses performed in this work were based on the generic Human Metabolic Reaction 2 (HMR2) genome-scale model [17]. GSMMs are an in silico representation gathering all the metabolic reactions encoded by an organism/tissue genome. More specifically, HMR2 model describes 8181 reactions, 3765 metabolic genes, 3160 unique metabolites and 136 biologic pathways. The model includes information about the stoichiometry of reactions, their reversibility, reaction substrate/products, associated pathways and/or enzyme activity. In addition, HMR2 presents a detailed annotation of lipid-associated metabolism compared with other reconstruction human metabolism, which makes this model especially well suited for lipidomic data integration.

Enabling GSMM for lipidomic and metabolic data integration: The model was modified to account for the consumption/production of the lipids and metabolites of interest. Important alterations in the lipidomic and metabolomic profile of DU145 cells have been associated to the chronic exposure to Aldrin [8]. Thus, the incorporation of the exchange reactions of the relevant lipids and metabolites has enriched our model with the specific features of the biological context under study. More specifically two irreversible reactions (exchange reactions), one up taken and other secreting, were added to all the species annotated in the model with relevant differences between groups. No new reactions were added if the exchange reaction already existed in the model. The model expansion has allowed determining the goodness of model predictions by comparing predicted metabolic consumption/production with experimental data (Additional file 4).

Reduction and Refinement: To eliminate those reactions in the model that cannot carry a flux different to zero (blocked reactions) [41] a reduction of the model was performed by applying the pruning function implemented in FASIMU software [42]. This function spots and removes those reactions that cannot carry a metabolic flux (flux equal to zero) in any condition. More specifically, if one reversible reaction has one of both directions blocked, it is turned into an irreversible reaction. In addition, once these reactions are removed, the species that no longer participate in any reaction, (dead-end metabolites) are removed from the model too (Additional file 4). The models used in this analysis are in Additional file 6.

### Stoichiometric gene-to-reaction association building

In this study we used one of the latest reconstructions of human metabolism [17], which does not incorporate GPRs. To enable the integration of transcriptomic data in this model, we developed an algorithm that extracts combines and interprets information retrieved from a variety of data bases [10–12, 43] to automatically build either GPRs or S-GPRs for each metabolic reaction with catalytic activity in the model. In brief the algorithm uses as input the enzyme commission code (EC) of each reaction and the sub-cellular location. The EC code classifies the enzymes based on the chemical reactions they catalyze [44]. Thus, each enzymatic reaction in the model is described by one EC code (or several in case the reaction describes a sequence of biochemical reactions catalyzed by a group of enzymes or a complex) that is associated to an enzyme, isoenzyme and/or complex that in turns is encoded by one or more genes. The sub-cellular location is inferred from the compartment of the metabolites involved in the reaction and it’s used to discriminate those genes encoding isoenzymes and/or complexes with the same catalytic activity but expressed in a different cellular compartment. Based on the EC code, the cellular compartment and the associated genes of a given reaction, the algorithm extracts, combines and interpret the information from different databases by applying Levenshtein automaton formalism [45] to generate a semantic tree that is used to build the corresponding S-GPR. Classical GPRs are generated by merely removing the stoichiometric information from the S-GPRs (Fig. 1B). The algorithm is written in Python programming language and can be found in the Additional file 5, the resulting GPRs and S-GPRs are described in the Additional file 4.

### Flux balance analysis

Flux Balance Analysis (FBA) [46] is one of the most used constraint-based modeling (CBM) methods in systems biology. Here, only stoichiometry and thermodynamic (reaction reversibility) information is required which makes this approach suitable to analyze large-scale metabolic networks such as GSMMs. This mathematical approach determines a space of feasible flux solutions that is consistent with the stoichiometric and thermodynamic constraints imposed by a given metabolic network. This space of feasible flux solutions can be further constrained by incorporating different high-throughput measurements such as transcriptomics. Finally, it is necessary to define a phenotype in the form of a biological objective that is relevant to the problem being studied, the objective function. This objective function is used to quantitatively define how much each reaction contributes to the chosen phenotype. Typically, this objective function is related to the growth rate which is defined by an artificial biomass production reaction [46]. In summary FBA enriched with transcriptomic data enables to determine an optimal metabolic flux profile that fits with a given phenotype which is described by an objective function. In this work FBA is enhanced by integrating transcriptomic data via GPRs and S-GPRs. This integration is performed through four different algorithms developed for this purpose [47] that are depicted in Fig. 1C.

### Transcriptomic data integration

Incorporating stoichiometry into gene-to-reaction associations: GPR and S-GPR design: GPR rules enable the association between gene expression levels and the activity state of the biochemical reactions in a GSMM. In brief, genes annotated in the gene-to-reaction associations are replaced by their corresponding expression levels (absolute or relative to a control depending on the used method). Next, the logical “OR”, representing different isoenzymes, is replaced by either “mean” or “maximum” operators, which varies depending on the approach, while the logical “AND”, representing an enzymatic complex, is replaced by the operator “minimum”. Here, we introduce a new state-of-the-art GPR: S-GPR, which take into account the number of transcript copies required to generate a fully functional protein that catalyzes a given reaction (Fig. 1B). Thus, for example the GPR of a reaction that is catalyzed by a complex with three subunits one encoded by the gene “a” and the two others by the gene “b” is: “a and b”, while the corresponding S-GPR is: “a and 2*b”.

Transcriptomic data integration through GPR and S-GPR associations: The integration of transcriptomic data into model-driven methods is based on the inference of metabolic reactions values by using the expression of the associated genes. Next, the reaction values are used to determine the activity state of a reconstructed metabolic network in a specific case/tissue/organism. The way in which transcriptomic data is propagated from genes to reactions and how reaction values are used to perform a metabolic network reconstruction analysis vary among the different integration methods. In the case of GPRs, the normalized gene expression is integrated with no previous modification, whereas in S-GPR, prior to the integration, the expression of each gene is divided by the number of needed copies described in the corresponding gene-to-reaction association. To determine the improvement at integrating stoichiometry into the classical GPR (S-GPR), transcriptomic data from Aldrin-exposed and non-exposed was integrated into a GSMM reconstruction analysis. To this aim four different algorithms were applied, each of them being representative of one of the main four approaches for integrating transcriptomic data into a constraint-based method (CBM) analysis (Fig. 1C).

#### Gimme

Gimme algorithm [20] integrates transcriptomic data of each experimental condition to build a metabolic network that satisfies the thermodynamic and stoichiometric constraints, while penalizing the inclusion of reactions catalyzed by genes with expression below a certain threshold. We proceed as it is indicated in [20]. Gimme was implemented in the computational environment provided by FASIMU [42]. Gimme classifies reactions as ON or OFF regarding their transcriptomics-based expression value whether it is over or under threshold, respectively. Thus, the higher the difference between the under-expressed reaction value and the threshold, the higher the penalty is (Fig. 2A). The chosen threshold was set at a value that maximizes the number of reactions that are ON in one condition and OFF in the other one. To this aim, different thresholds were evaluated (Additional file 9.II and Additional file 8).

#### iMat

iMat method [19] assigns to the metabolic reactions a discrete state (low, moderate or high) based on the expression of the associated genes in a specific experimental condition. Next, it seeks a stoichiometric and thermodynamically consistent steady-state flux solution while maximizing the number of active reactions associated with highly expressed genes and minimize the number of active reactions associated to lowly expressed genes. It was proceeded as indicated in [19]. To determine whether a gene is over or under-expressed it is necessary to define an upper and a lower threshold (Fig. 2B). In this study, the chosen thresholds were those that provided the highest difference between conditions (Additional files 9 and 8).

#### Gonçalves et al. 2012

The method proposed by Gonçalves et al. 2012 [21], uses relative gene expression values from experimental conditions and integrates them as continuous expression levels. This method uses treated gene expression levels relative to a control to define the upper and lower bounds of the metabolic reactions. In a first step, it is performed a parsimonious FBA (pFBA) [48] which is a two-step variant of FBA and defines the metabolic flux profile in control group. Next, gene expression of Aldrin-exposed cells relative to control is integrated through either GPR or S-GPR. As it is depicted in Fig. 2C, there can be two cases when classifying reactions into over-activated or under-activated. The boundaries of the reactions are modified depending on the product between the relative expression and the control flux of a concrete reaction and its relationship with the control flux itself. From that set of inequalities, the method proposed by Gonçalves et al. 2012 creates a new set of relative-expression-based boundaries for the reactions of the model. Finally, another pFBA is performed for the relative expression data sets.

#### Made

MADE algorithm [22] uses gene expression values relative to a control and integrates them as discrete expression levels. This method uses gene expression levels to determine whether a reaction is up, down-activated or has the same activity in one condition relative to a control. These discrete levels are set by using the log fold change of the reactions based on the associated genes and the corresponding *p*-value. Next, the algorithm finds a solution that is consistent with maximum number of relative discrete levels while is constrained by the stoichiometry and thermodynamics imposed by the model. In other words, if a given reaction is up-regulated in Aldrin-exposed cell the algorithm will be penalized if provides a solution in which the reaction is active in control and inactive in Aldrin-treated cells (Fig. 2D).

### Using metabolic and lipidomic data to evaluate the improvement of using S-GPR

The different computational methods to integrate transcriptomic data via either GPR or S-GPR predicted the metabolism in non-exposed and Aldrin-exposed cells (metabolite consumption/production). In order to assess the reliability of the model predictions and to determine the improvement associated to the use of S-GPR, a qualitative comparison is performed between the predicted metabolic consumption/productions and the metabolomic and lipidomic experimental measurements. A Fisher exact test is applied for this purpose [23] (Fig. 1 E and F and Additional file 8).

Metabolic pathway analysis to unveil the metabolic alterations associated to the chronic exposure to endocrine disruptor in Prostate Cancer.

The phenotype differences associated to the chronic exposure to Aldrin in DU145 cells should be reflected in a different activity state of the metabolic reactions between Aldrin-exposed and non-exposed cells. Thus, these reactions are of interest to understand the metabolic reprogramming underlying the enhancing of malignancy associated to the chronic exposure to ED in PC. To this aim, a pathway analysis is performed based on the reactions that are consistently predicted to have different activity state between control (or non-exposed cells) and Aldrin-exposed cells with all the methods used in this work. More specifically, each metabolic reaction is associated to a given metabolic pathway in the GSMM, which enables to determine whether a particular pathway is over or under-active between conditions using the number of active reactions in each condition. To this aim, the following procedure was used: All the reactions which were stated as differentially active in both experimental conditions from the results of most of the different transcriptomics-based model-driven analysis were selected. Next, all pathways from these reactions were tracked down to have a list of the most active pathways (pathways with more active reactions). This analysis was performed in each integration analysis for both experimental conditions. The significance was determined by a t-test analysis. Finally, a bibliographic research was performed in order to find evidences that support these findings.

## Additional files

Additional file 1: Metabolomic and Fluxomic experimental data. Metabolomic data and ROI analysis result from Aldrin-exposed and non-exposed DU145 cells at time 0 and after 5 h. (XLSX 1253 kb)

Additional file 2: Mapping metabolomic and lipidomics on GSMMs: Metabolomic and Lipidomic data mapped on HMR2 and Recon2 models. (XLSX 296 kb)

Additional file 3: Transcriptomic data. Transcriptomic data of Aldrin-exposed and non-exposed DU149 PC cells and gene expression analysis. (XLSX 4835 kb)

Additional file 4: GSMM readjustments/refinement process. This file includes; i) Definition of Metabolites included in the biomass reaction, ii) Required Metabolic Functionalities (RMF) necessary to apply GIMME algorithm, iii) Experimentally measured metabolites annotated in HMR2 and iv) automatically generated GPR and S-GPR of each catalytic reaction in HMR2 (algorithm in Additional file 5) (XLSX 190 kb)

Additional file 5: Gene-to-reaction building algorithm: Algorithm to build GPR and S-GPR (written in Python). (ZIP 2623 kb)

Additional file 6: GSMMs: Genome-scale metabolic models used in the analysis. (ZIP 2743 kb)

Additional file 7: Comparison of the reliability of model predictions between HMR2 + algorithm-based GPRs and Recon2. (XLSX 501 kb)

Additional file 8: Results of the analyses comparing predictive capabilities of GPR-based and S-GPR-based approaches using iMat, Gimme, Gonçalves et al. 2012 and MADE algorithms. (XLSX 309 kb)

Additional file 9 Supplementary methods and further biological interpretation. (DOCX 15 kb)

## Data Availability

All data generated or analyzed during this study is included in this published article [and its supplementary information files]. In addition, transcriptomic data is deposited in GEO database (GSE132063- https://www.ncbi.nlm.nih.gov/geo/query/acc.cgi?acc=GSE132063).

## Abbreviations

CBM: Constraint-based modeling
EC: Enzyme commission
ED: Endocrine disruptor
FBA: Flux Balance Analysis
GPR: Gene-protein-reaction
GSMM: Genome-scale metabolic models
HMR2: Human Metabolic Reaction 2
MCR-ALS: Multivariate curve resolution alternating least squares
PC: Prostate Cancer
pFBA: Parsimonious FBA
ROI: Region of interest
S-GPR: Stoichiometric Gene-protein-reaction
STNRT: Signal-to-noise ratio threshold
