# Supplementary material for: Stoichiometric gene-to-reaction associations enhance model-driven analysis performance: Metabolic response to chronic exposure to Aldrin in prostate cancer
Source: BMC Genomics. 2019 Aug 15;20:652. doi: 10.1186/s12864-019-5979-4 (PMC6694502; doi:10.1186/s12864-019-5979-4)
Supplement: Supplementary file 9 — Supplementary methods and further biological interpretation. (DOCX 15 kb) [file 12864_2019_5979_MOESM9_ESM.docx]

**SUPPLEMENTARY MATERIAL**

**I. Gene-to-reaction associations automatic building**

The algorithm for automatic GPR and S-GPR building is written in python language and runs under python 2.7. The script is divided in three main parts: i) definition of the classes and the corresponding attributes, ii) definition of the functions and iii) the routine where the previously defined objects and functions are invoked to build the gene-to-reaction associations.

The algorithm uses two types of inputs that are extracted from the sbml file describing the metabolic model: i) the enzyme commission number (EC) associated to every reaction and the compartment of the metabolites involved in the reaction and ii) the cellular compartment of the metabolites involved in each (mitochondria, cytosol, peroxisome, …).

The process starts by extracting and matching information about the enzyme activity (EC number), and the subcellular location (based on the compartment of the metabolites involved in the reaction). The EC number is used to determine the associated genes by using information retrieved from KEGGand BioCyc databases. Next the list of genes is compared with NCBI and Uniprot databases to determine the subcellular location of each gene and thus choose the ones that are consistent with the subcellular location of the metabolites involved in the reaction. Based on this information, the algorithm finds the gene-to-reaction (GPR) associations retrieved from BioCyc, this database also incorporates information about transcript copy number and the number of subunits required for each metabolic reaction. If no information is found in BioCyc, the algorithm uses KEGG database or Recon 2 if KEGG have no information about a given enzymatic activity (typically for more general EC numbers like 1.1.-.-).

The algorithm is able to read, interpret and modify the information retrieved in the different databases to generate gene-to-reaction associations with the right format and properly nested. For example, the algorithm can read sentence like “two subunits encoded by the gene X are required ...” and transform it into “2*X”. The algorithm is also able to determine if there is more than one isoenzyme in a cellular compartment, then algorithm integrates both isoenzyme genes in a single S-GPR and connect them through the logical “or” or determine complexes in which case the genes are connected through the logical “and”. The GPRs are generated by merely removing the stoichiometric information from the GPRs. Finally the algorithm generates a new SBML file describing a metabolic model that incorporates either S-GPRs or GPRs

**II. Evaluating the accuracy of the gene-to-reaction automatic building algorithms**

In order to evaluate the reliability of our algorithm at building gene-reaction associations in the context of transcriptomic-based model-driven methods, we compared the metabolite uptake/secretion predictions provided by HMR2 [1] model with automatically-build GPRs with Recon2 [2] with manually curated GPRs. This analysis enables to evaluate if the predictions provided by models incorporating automatically build gene-to-reaction associations have similar reliability than predictions from existing models incorporating GPRs. In consequence, the results of this analysis provide the basis by which the subsequent incorporation of stoichiometry into GPRs can be evaluated

This analysis was performed in two steps:

**1. Determine flux spectrum profile of Aldrin-exposed and non-exposed PCa cells by Flux variability analysis:** We determine the flux spectrum profile of Aldrin-exposed and non-exposed cells by integrating transcriptomic data into a GSMM reconstruction based on Flux Variability analysis (FVA) [3-5]. FVA computes the spectrum of fluxes that each reaction can carry while the value of the objective function is optimal. Thus, it determines the minimal and maximal flux through each reaction. This approach is carried out in two steps. Firstly, we determine the potential active reactions by integrating transcriptomic data followed by a flux balance analysis (FBA). Transcriptomic data is integrated by using iMat algorithm [6]. As a result we obtain a set inactive reaction (carrying flux 0) of potentially active reactions. Next, the quantitative lipidomic data (experimentally determined upper and lower bounds of corresponding exchange reactions) is integrated and FVA is employed to determine the flux spectrum for each reaction. As a result, the minimum and maximum flux values are obtained for each reaction.

**2. Comparing predicted and experimental metabolite consumption/production:** In order to determine the reliability of our model predictions we compared the predicted metabolite consumption/production with the experimental observations. The validation part is based on statistical comparison of model-predicted FVA flux values with that of experimentally obtained. The obtained FVA flux values were validated by using metabolomic and lipidomic experimental data of 38 metabolites and 17 lipid species. The corresponding FVA values of exchange and sink reactions for these metabolites and lipid species are extracted from FVA data results and compared with experimental data. The contingency table was created for reaction directionality prediction accuracy of model-predicted exchange and sink reactions upper and lower bounds compared to that of experimental determined. This comparison provided numbers of hits for true positive, true negative, false positive and false negative results of reaction directionality which are ordered in 2x2 contingency table on which the chi square statistical test is performed.

As a result these analyses revealed that the HMR2 model incorporating the GPRs generated by our algorithm provided better predictions than Recon2 model in both Aldrin-exposed and non-exposed group, with an overall improvement of 31%. These results are shown in more detail in the Supplementary material 7.

**III. Thresholds choice: Gimme and iMat**

Both Gimme and iMat algorithms require predefined threshold/s to determine whether a given gene is up or down-regulated [6,7]. Gimme algorithm defines a single threshold to separate up-regulated genes (above the threshold) and down-regulated genes (below the threshold) while iMat method defines two thresholds to differentiate up-regulated (above upper threshold), down-regulated (below lower threshold) and moderately expressed (between thresholds). In this study we choose a threshold/s that maximize the categoric difference between Aldrin-exposed and non-exposed cells. In other words, maximize the number of reactions considered as associated to up-regulated genes in one condition and down-regulated genes in the other. To this aim different thresholds were evaluated as follows: in each condition, the up-regulated reactions were set as 1 while the down-regulated were set as 0 resulting in boolean vector. Next the vectors corresponding to Aldrin-treated and non-treated cells were compared resulting in a new boolean vector of the same length were 0 and 1 represents genes with the same or different categorical classification respectively. Finally, a score was calculated by summing all the elements and dividing by the length of the vector. This score quantifies the categorical difference in the activity state of the reactions between Aldrin-exposed and non-exposed cells. Thus, the higher the score is, the more differences are between conditions. This process was performed to evaluate different thresholds in each methods (Table S1). Here, in iMat, lower and upper thresholds were set at 40^th^ and 60^th^ percentiles respectively, while the chosen threshold in Gimme algorithm was the median + standard deviation.

**Table S1.** Thresholds evaluation for Aldrin-exposed (AE) and non-exposed (N-AE) DU145 cells. A: Thresholds evaluated in iMat algorithm and the corresponding scores. UT and LT correspond to upper and lower thresholds values respectively. B: Threshold evaluated in Gimme algorithm and the corresponding scores. T is the evaluated threshold value. These results are in more detail in the Supplementary material 8.

**IV-Pathways analyses and biological interpretation**

Nine biological pathways were stated as over-activated in Aldrin-exposed analyses. Even though only two of them are common among all four methods, all the pathways are coherent with the biological context of our case study and have bibliographic support:

i) Prostaglandin Biosynthesis pathway has already been described as a prostate-cancer-related pathway before [8]. The lipids related to this pathway are involved in the regulation of mechanisms associated to malignancy.

ii) The pentose phosphate pathway is a key pathway to keep redox homeostasis to prevent oxidative stress and it is activated when the cell is under oxidative stress condition [9]. Not only was it a regulation control point, but also it keeps the NADP(H) balance and the production of glucose by up-regulating an important glucose-production related enzyme [10]. It is important to mention that one typical phenotype from a cancer cell is the increased demand of glucose because of the Warburg effect [11].

iii) Leukotrien metabolism is usually over-activated in different kind of cancers. It is usually related to an increase of the amount of pro-inflammatory cells. Moreover, it is widely agreed that chronic tissue inflammations lead to an increase of the risk of cancer, or that it leads to a progression of an

existing cancer [12].

iv) Glicosphingolipid Biosyntesis / Lacto and NeoLacto series are related to glycan epitopes that can be found in blood group antigens and they are related to malignant disease, for example in ovarian cancer, in which they found that these kinds of glycosphingolipids are implied in a glycosylation complex [13].

v) and vi) Both Carnitine shuttles, endoplasmatic reticular and endoplasmatic mitochondrial ones, are over activated in cancer phenotypes due to that the need of energy because of the Warburg effect. The aberrant lipid phenotype in prostate cancer may lead to different lipid metabolism and mechanisms in both ER and Mitochondria [14].

vii) Arginine and Proline pathway activation fits with previous work which claimed that arginine

metabolisation is androgen-regulated in human prostate cancer [15]. Moreover, both proline and

arginine are metabolically linked to the production of glutamine, the second most important substrate for tumors after glucose [16]. Also, both arginine and proline metabolites were significantly produced in Aldrin-exposed cells in the experimental analysis.

viii) High retinol concentration in serum is correlated to a greater chance to have prostate cancer over the years [17]. Even though retinol was not spotted as significantly produced in the experimental analysis, vitamin D3 was significantly consumed. Regarding bibliography [18], high vitamin D3 concentration would down-regulate retinol pathway activity. Thus, being vitamin D3 significantly consumed in the results fit with the expected biological behavior.

ix) Pyruvate pathway is over-active in Aldrin-exposed cells analysis. Metabolites from the pentose phosphate pathway end up taking part in glycolysis producing pyruvate. In addition, in general in cancer cells the gene of pyruvate kinase which is expressed is PKM2. PKM2 is also overexpressed in Aldrin-exposed cells in the transcriptomic analysis and moreover a reaction that has been predicted by our computational analyses in which this gene takes part is also over-activated in Aldrin-expressed condition.

**Supplementary references**

1. Pornputtapong N, Nookaew I and Nielsen J., "Human metabolic atlas: An online resource for human metabolism," *Database (Oxford)* 2015:1–9, 2015.
2. Thiele I *et al.*, "A community-driven global reconstruction of human metabolism," *Nat Biotechnol.* 31(5):419-25, 2013.
3. Llaneras F and Pico J, "A procedure for the estimation over time of metabolic fluxes in scenarios where measurements are uncertain and/or insufficient," *BMC Bioinformatics* 8:421, 2007.
4. Jennifer L Reed and Bernhard Ø Palsson, "Genome-scale in silico models of e. coli have multiple equivalent phenotypic states: assessment of correlated reaction subsets that comprise network states," *Genome Res.* 14(9):1797–1805, 2004.
5. Mahadevan R and Schilling C H, "The effects of alternate optimal solutions in constraint-based genome-scale metabolic models," *Metab Eng.* 5:264–276, 2003.
6. Zur H, Ruppin E. and Shlomi T., "iMAT: An integrative metabolic analysis tool," *Bioinformatics*. 26(24):3140–2, 2010.
7. Becker SA and Palsson BO., "Context-specific metabolic networks are consistent with experiments," *PLoS Comput Biol.* 4(5), 2008.
8. Badawi a F., "The role of prostaglandin synthesis in prostate cancer," *BJUInt.* 85:451–62, 2000.
9. Stincone A. *et al.*, "The return of metabolism: biochemistry and physiology of the pentose phosphate pathway," *Biol Rev Camb Philos.* 90(3): 927–963, 2015.
10. Tsouko E *et al.*, "Regulation of the pentose phosphate pathway by an androgen receptor–mTOR-mediated mechanism and its role in prostate cancer cell growth," *Oncogenesis.* 3(5):e103. 2015
11. Deep G and Schlaepfer IR., "Aberrant lipid metabolism promotes prostate cancer: Role in cell survival under hypoxia and extracellular vesicles biogenesis," *Int J Mol Sci.* 17(7), 2016.
12. Dubois RN., "Leukotriene A 4 Signaling , Inflammation , and Cancer," *Cancer* 95(14):4–5, 2003.
13. Drake RR, Jones EE, Powers TW and Nyalwidhe JO., "Altered glycosylation in prostate cancer," *Adv Cancer Res.* 126:345-82, 2015.
14. Deep G. and Schlaepfer IR., "Aberrant lipid metabolism promotes prostate cancer: Role in cell survival under hypoxia and extracellular vesicles biogenesis," *Int J Mol Sci.* 17(7), 2016.
15. Gannon PO *et al.*, "Androgen-regulated expression of arginase 1, arginase 2 and interleukin-8 in human prostate cancer," *PLoS One.* 11;5(8):e12107, 2010.
16. Phang JM and Liu W., "Proline metabolism and cancer," *Front Biosci.* 17, 1835-1845, 2012.
17. Mondul AM *et al.*, "Serum retinol and risk of prostate cancer," *Am J Epidemiol.* 173(7):813–21, 2011
18. Törmä H, Rollman O, Binderup L and Michaelsson G., "Vitamin D analogs affect the uptake and metabolism of retinol by human epidermal keratinocytes in culture," *J Investig Dermatol Symp Proc.* 1(1):49-53, 1996.
